# Magnitude of Anemia and Its Associated Factors Among Pregnant Women Attending Antenatal Care in Hiwot Fana Specialized University Hospital in Eastern Ethiopia

**DOI:** 10.3389/fpubh.2022.867888

**Published:** 2022-05-30

**Authors:** Bikila Balis, Yadeta Dessie, Adera Debella, Addisu Alemu, Dawit Tamiru, Belay Negash, Habtamu Bekele, Tamirat Getachew, Addis Eyeberu, Sinetibeb Mesfin, Bajrond Eshetu, Bedasa Taye Merga, Sisay Habte, Tesfaye Assebe Yadeta

**Affiliations:** ^1^School of Nursing and Midwifery, College of Health and Medical sciences, Haramaya University, Dire Dawa, Ethiopia; ^2^School of Public Health, College of Health and Medical sciences, Haramaya University, Dire Dawa, Ethiopia

**Keywords:** Anemia, magnitude, associated factors, pregnant women, Ethiopia

## Abstract

**Background:**

Anemia is a significant public health issue, accounting for 20–40% of maternal deaths. Despite the government's commitment and the interventions of various stakeholders, the magnitude and major risk factors of anemia remain unabated. Though there are few documented studies on anemia among pregnant women in eastern Ethiopia in general, in the study area in particular, some of the variables such as helminthics and history of caesarian section in relation to anemia need to be studied. As a result, the purpose of this study was to determine the magnitude of anemia and associated factors among pregnant women attending antenatal care in University Hospital in eastern Ethiopia.

**Methods:**

A facility-based cross-sectional study was conducted on a sample of 456 clients who were attending antenatal care in Hiwot Fana specialized university hospital from 01 to 30 June 2021. Systematic sampling was used to select the study participants. A pretested and structured interviewer-administered questionnaire and sample collection were used to collect the data. The data were coded, double-entered to Epi data version 3.1, cleaned, and exported to SPSS version 20 for analysis. Descriptive statistics were used to present frequency distributions. Variables with *p*-value < 0.25 during bivariate analysis were entered into the multivariate logistic regression models to control for all possible confounders to identify the factors associated with a magnitude of anemia. Odds ratio along with 95% CI were estimated to measure the strength of the association. The level of statistical significance was declared at a *p*-value of < 0.05.

**Result:**

A total of 456 participants were interviewed, with a response rate of 96.9%. The magnitude of anemia among pregnant women was 112 [(25.3%) 95%CI: (21.5–29.2%)], of which, 27 (6.10%), 36 (8.13%), and 49 (11.08%) had mild, moderate, and severe anemia, respectively. Birth interval < 2 years [AOR: 3.24, (95% CI: (1.88, 4.32)], number of children ≥2 [AOR: 2.54, (95% CI: (1.12, 4.64)], monthly income < 1,000 birr [AOR: 2.89, (95% CI: (1.31, 5.58)], third trimester pregnancy [AOR: 2.89, (95% CI: 4.86, 12.62)], and abnormal menstrual history [AOR: 2.28, (95% CI: (1.69, 5.24)] were the factors significantly associated with anemia.

**Conclusion:**

Anemia among pregnant women was relatively high compared to previous studies. Birth intervals, number of children, history of menstrual disorder, monthly income, and trimester of pregnancy were all significantly associated with anemia in pregnant women.

## Introduction

Anemia is a serious global public health problem that affects 40% of pregnant women (1). Anemia is a condition in which the hemoglobin concentration required to carry oxygen in venous blood is < 11 g/dl in the first and third trimesters and 10.5 g/dl in the second trimester (2, 3). To prevent anemia, the WHO recommends to take 60 mg of iron and 400 g of folic acid daily during the last 6 months of pregnancy. Despite this, more than 56 million pregnant women suffer from anemia worldwide, with Africa accounting for two-thirds (17.2 million) (4).

Similarly, numerous evidences show that a large number of pregnant women in Ethiopia suffer from varying degrees of anemia, ranging from 9.7 to 56.8% (5–23). Untreated anemia in pregnant women has severe consequences for the nation's social and economic development in addition to perinatal and maternal morbidity (4, 24).

A large number of women from low-income countries begin their pregnancies with frank iron deficiency anemia (IDA) and/or depleted iron stores, which accounts for 90% of anemia (25) and 20–40% of maternal deaths (26, 27). In Ethiopia, maternal mortality remains too high, with a maternal mortality rate of 412 per 1,00,000 live births each year (28). Furthermore, IDA is associated with perinatal morbidity and mortality, as well as long-term adverse effects in newborns (25), such as growth restriction, low birth weight, and preterm birth (25, 28).

Many studies in Ethiopia have found that teenage pregnancy, education, monthly income, residence, frequency of meal, family size, trimester of pregnancy, menstrual disorder, infection, and birth interval are all the risk factors for anemia during pregnancy (6, 7, 14, 20, 23, 29).

According to the Ethiopian Demographic and Health Survey (EDHS), the prevalence of anemia among women aged 15–49 years fell from 27 to 17% in 2011, but increased to 24% in 2016, with pregnant women accounting for 29% (30). The national figures from the EDHS, however, could not represent the prevalence of anemia among pregnant women in different parts of Ethiopia due to the differences in socioeconomic status, behavioral, geographical, and methodological differences.

Despite the government's commitment and the efforts of various stakeholders, the magnitude and major risk factors of anemia persist. Though there are few documented studies on anemia among pregnant women in eastern Ethiopia in general, in the study area in particular, some of the variables such as helminthics and history of caesarian section in relation to anemia need to be studied. As the result, this study has aimed to determine the magnitude of the anemia and its associated factors among pregnant women in university hospital, in eastern Ethiopia.

## Methods and Materials

### Study Setting, Design, and Period

A facility-based cross-sectional study was conducted in Hiwot Fana Specialized University Hospital (HFSUH) in Harari regional state, 526 km from Addis Ababa from 01 to 30 June 2021. The hospital is found in Harar, the capital city of Harari region. According to the 2007 Statistical Report of the Ethiopian Population and Housing Census, the region has a total population of 1,83,415 people, with 92,316 men and 91,099 women (31). The hospital is one of the main academic referral centers in eastern Ethiopia, serving a population of over 5 million people. The study was conducted in the obstetrics department. The department was run by seven consultants, 32 residents, and 243 midwives and/or nurses during the study period. According to hospital records, 10,000 pregnant women attend ANC follow-up each year.

### Source Population, Study Population, and Eligibility Criteria

All pregnant women who were attending antenatal care (ANC) were included in the study. A sample of pregnant women who were attending ANC for the current pregnancy was the study population. Women who have confirmed pregnancy and attended their ANC visit during study period were included in the study. Women who came with bleeding (PPH), were unable to communicate, were severely ill, had a known diagnosis of anemia, and were taking iron, as well as women who were undergoing invasive or non-invasive anemia treatment, were excluded.

### Sample Size Determination

The sample size was determined based on the predictors of women residence, using statistical Epi info 7 stat calculator computer software program using 95% CI with Z = 1.96, cross-sectional ratio of 1:1, the proportion of urban resident (31.6%) and rural resident (41.9%), respectively (14), and 80% power and 5% non-response (*n* = 456). Since sample size calculated for residence in Gebre and Mulugeta (14) was the highest, it was taken as the sample size for this study (Table 1).

**Table 1:** Sample size calculation to identify the factors associated with anemia among pregnant women attending ANC in a University hospital in eastern Ethiopia, 2021.

**Factors**	**Assumptions**	**Sample size**	**Sample size + non-response (5%)**	**References**
Intestinal parasite infection	CI: 95% Power: 80% Ratio: 1:1 Exposed (Yes): 50.5% Unexposed (No): 30.9%	216	227	(7)
Residence	CI: 95% Power: 80% Ratio: 1:1 Exposed (rural): 41.9% Unexposed (urban): 31.6%	434	456	(14)
Contra ceptive use	CI: 95% Power: 80% Ratio: 1:1 Exposed (No): 61.6% Unexposed (Yes): 35.6%	130	137	(29)

### Sampling Techniques

A systematic sampling method was used to obtain 456 participants. The sampling interval was calculated by dividing the number of women who attended ANC (*n* = 1,024) at HFSUH in the previous month by the total sample size (*n* = 456). The skipping interval for participant selection was calculated by dividing the total population (*n*=1,024) by the estimated sample size (*n* = 456), yielding a kth interval of two. As a result, all participants were obtained at 2-week intervals after the first participants were obtained through the lottery method.

### Blood Sample Collection and Examination

A portable heme analyzer (HemoCue 301 Hb; HemoCue, Angelholm, Sweden) was used to measure hemoglobin levels. The non-dominant hand's middle finger was pricked at the side of the fingertip. A drop of blood was drawn and placed on a micro-cuvette before being inserted into the heme analyzer. Hemoglobin levels were read and recorded to one decimal place after the machine was calibrated. The packed cell volume was calculated by dividing the hemoglobin values (in g/dl) by three and then subtracting the units.

### Data Collections Tools and Procedures

A questionnaire was adapted from a different literature (13, 15, 17, 19–22, 32–35) and was designed to obtain participant information on sociodemographic characteristics, obstetric and gynecologic characteristics, food intake characteristics, and disease and infection characteristics. The questionnaire was initially developed in English and the translated into the local languages (Afaan Oromo and Amharic) before being translated back to English. Statistically, we performed Cronbach's alpha, which is a measure used to assess the quality of our employed instruments. The result was 0.87, which was within acceptable ranges. Data were collected by interviewer-administered questionnaires by five midwives, and specimen collection and processing were carried out by two trained laboratory technologists. The collection, processing, and analysis of specimens were supervised by skilled and trained laboratory technicians as well as trained health professionals with research experience.

### Measurement

Anemia was assessed by measuring hemoglobin level during the study period. Hemoglobin levels <11g/dl in the first and third trimesters and 10.5g/dl in the second trimester were considered anemia, and the severity of anemia in pregnancy was classified into three categories according to WHO criteria: mild (10.0–10.9 g/d1), moderate (7–9.9 g/dl), and severe (7 g/dl)(36).

### Data Quality Control

To ensure data quality, an appropriate data collection instrument was developed. Trained data collectors were regularly supervised to ensure proper data collection; all questionnaires were checked daily for completeness and consistency. A pretest was conducted on 5% of the sample size in a nearby public hospital that was not one of the main study's recruitment sites, after which the questionnaire was revised, edited, and any questions found to be unclear or ambiguous were removed or corrected accordingly. All laboratory procedures were carried out in accordance with standard operating procedures.

### Data Processing and Analysis

The data were coded, double-entered into Epi data version 3.2, cleaned, and exported to SPSS version 20 for analysis. The Hosmer–Lemeshow goodness-of-fit test was used to determine the correlation between independent variables, and the multi-collinearity test was used to determine the correlation between independent variables (0.86). Descriptive statistics were used to present frequency distributions. Bivariate analysis was employed to identify the factors associated with magnitude of anemia. Variables with *p*-value <0.25 during bivariate analysis were entered into the multiple logistic regression models to control for all possible confounders to identify the factors associated with magnitude of anemia. Odds ratio along with 95% CI were estimated to measure the strength of the association. Level of statistical significance was declared at a *p*-value <0.05.

## Results

### Sociodemographic Characteristics

In this study, a total of 442 respondents were interviewed out of 456, with a response rate of 96.9%. The majority of respondents, 250 (56.6%), were between the ages of 19 and 25. More than half of the respondents, 234 (52.9%), were Oromo by ethnicity, and 220 (49.8%) were unemployed. In terms of marital status, 415 (93.9%) of respondents were married, and 234 (52.9%) earned more than 2,000 birr per month. More than six out of 10 respondents, 276 (62.4%), had no or one child, and more than three-quarters, 344 (77.8%), resided in urban centers (Table 2).

**Table 2:** Socio-demographic characteristics of respondents attending ANC in a University Hospital in Eastern Ethiopia, 2021 (*n* = 442).

**Characteristics**	**Category**	**Frequency**	**Percent (%)**
Age	≤ 18 years	37	8.4
	19–25 years	250	56.6
	26–30 years	130	29.4
	≥ 31 years	25	5.7
Marital status	Single	6	1.4
	Married	415	93.9
	Divorced	14	3.3
	Separated	7	1.6
Religion	Muslim	218	49.3
	Orthodox	142	32.2
	Protestant	73	16.5
	Others^*^	9	2.0
Ethnicity	Oromo	234	52.9
	Amhara	116	26.3
	Harari	57	12.9
	Others^**^	35	7.9
Occupation	Housewife	220	49.8
	Government employees	51	11.5
	Marchant	128	29
	Daily laborer	23	7.5
Number of children	≤ 1 child	276	62.4
	≥ 2 children	166	37.6
Educational level	Not educated	45	10.2
	Primary school	249	56.3
	Secondary and above	148	33.5
Monthly income	<1000 birr	93	21.0
	1000–2000 birr	115	26.1
	≥ 2001 birr	234	52.9
Residence	Urban	344	77.8
	Rural	98	22.2

* *Catholic*,

** *Somale, Tigre, Gurage*.

### Obstetric and Gynecologic Characteristics

More than two-thirds, 302 (68.3%), of respondents had ≥2 number of pregnancy, and three-fourths, 222 (76.0%), of respondents were wait for ≥2 years for next pregnancy. During the interview, 317 (71.7%) of the total respondents were found to be in their second trimester. In terms of menstrual cycle, 412 (93.2%) of respondents had a regular menstrual history. In terms of obstetric history, 17(3.8%), 8(1.8%), 20(4.5%), and 15(3.4%) had cesarean section, preeclampsia and/or eclampsia, antepartum, and postpartum hemorrhage, respectively (Table 3).

**Table 3:** Obstetrics and gynecologic characteristics of respondents attending ANC in a University hospital in Eastern Ethiopia, 2021 (*n* = 442).

**Characteristics**	**Category**	**Frequency**	**Percent (%)**
Gravida	0–1	140	31.7
	≥ 2	302	68.3
Birth interval	<2 years	70	24.0
	≥2 years	222	76.0
Trimester of pregnancy	1^st^ trimester	77	17.4
	2^nd^ trimester	317	71.7
	3^rd^ trimester	48	10.9
History of abnormal menses	Yes	30	6.8
	No	412	93.2
History of abortion	Yes	75	17.0
	No	367	83.0
Contraceptive use	Yes	147	33.3
	No	295	66.7
History of antepartum hemorrhage	Yes	17	3.8
	No	367	96.2
History of post-partum hemorrhage	Yes	8	1.8
	No	434	98.2
History of cesarean section	Yes	20	4.5
	No	422	95.5
History of pre/eclampsia	Yes	15	3.4
	No	427	96.6

### Dietary Intake Characteristics

In terms of food intake, more than three-fourths of participants, 346(78.3%), eat three times per day, and the most commonly consumed food types were injera [291 (65.8%)], bread [83 (18.8%)], and vegetables and fruits [26 (5.9%)]. Similarly, nearly three-fourths of respondents, 328 (74.2%), drank coffee or tea within 30 min of finishing a meal. Of the total respondents, 200 (45.2%) and 242 (54.8%) eat animal products (meat, egg) one time and ≥2 times per week, respectively. Also, 324 (73.3%) pregnant women eat vegetables and fruits ≥2 times per week (Table 4).

**Table 4:** Dietary intake characteristics of respondents attending ANC in a University hospital in Eastern Ethiopia, 2021 (*n* = 442).

**Characteristics**	**Category**	**Frequency**	**Percent (%)**
Drink coffee/tea within 30 min	Yes	328	74.2
	No	114	25.8
Chewing Kchat	Yes	85	19.2
	No	357	80.8
Frequency of eating animal products (meat, egg) per week	Once	200	45.2
	Two or more	242	54.8
Frequency of eating vegetables and fruits per week	Once	118	26.7
	Two or more	324	73.3
Eat meal at least three per day	Yes	346	78.3
	No	96	21.7
Commonly used food	Injera	291	65.8
	Bread	83	18.8
	Vegetables and fruits	26	5.9
	Others^*^	42	9.5

* *Biscuit, meat, egg*.

### Disease and Infection Characteristics

In terms of disease and infection history, 15 (3.4%), 9 (2.0%), and 27 (6.1%) pregnant women had malaria, chronic disease (diabetes mellitus, hypertension, HIV, cardiac or renal diseases), and intestinal helminths, respectively. In addition, 27 (6.1%) of the participants were dewormed (Table 5).

**Table 5:** Diseases and infection characteristics of respondents attending ANC in a University hospital in Eastern Ethiopia, 2021 (*n* = 442).

**Characteristics**	**Category**	**Frequency**	**Percent (%)**
History of intestinal helminths	Yes	27	6.1
	No	415	93.9
History of dewormed	Yes	27	6.1
	No	415	93.9
History of malaria	Yes	15	3.4
	No	427	96.6
Chronic diseases (DM, HTN, HIV, cardiac or renal diseases)	Yes	9	2.0
	No	433	98.0

### Magnitude of Anemia

**Figure 1:**
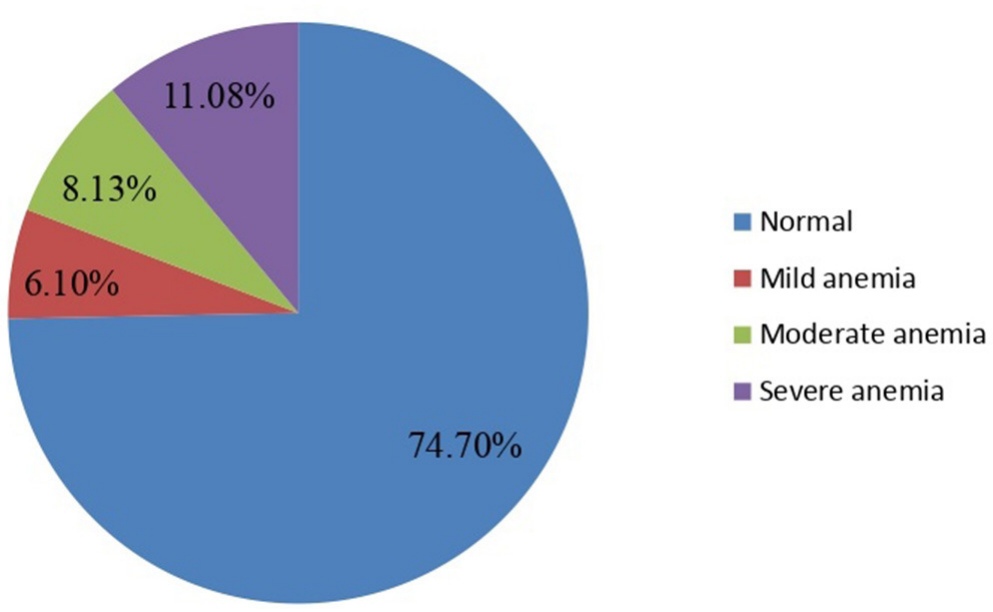
Magnitude of anemia among pregnant women attending ANC in a University hospital in Eastern Ethiopia, 2021 (*n* = 442).

Nearly one-fourth of the respondents, 112 [(25.3%) 95% CI: 21.5–29.2%], were anemic. Anemia was found in 27.10% of pregnant women, 36.13, and 49.08% of pregnant women with mild, moderate, and severe anemia, respectively (Figure 1).

### Anemia and Its Associated Factors

In bivariate logistic regression, family size, residence, educational level, monthly income, birth interval, gravida, gestational age, menstrual disorder, history of antepartum hemorrhage, history of postpartum hemorrhage, history of cesarean section, history of abortion, drink coffee or tea within 30 min, and chewing chat were the factors significantly associated with anemia.

However, in multiple logistic regression, respondents who stay for < 2 years between each pregnancy were nearly three-folds [AOR: 3.24, (95% CI: (1.88, 4.32))] more likely to have anemia than their counterpart. Similarly, women who had ≥2 children were 2.54 times [AOR: 2.54, (95% CI: (1.12, 4.64))] more likely to develop anemia than women who have ≤1 child. Also, women who have monthly income < 1,000 birr were almost three times [AOR: 2.89, (95% CI: (1.31, 5.58)] more likely to have anemia than their counter parts. Women with third trimester gestational age were nearly four times [AOR: 2.89, (95% CI: (4.86, 12.62))] more likely to experience anemia compared to first trimester. Similarly, women who have history of abnormal menstrual history were two times [AOR: 2.28, (95% CI: (1.69, 5.24)] more likely to have anemia than their counter parts (Table 6).

**Table 6:** Factors associated with anemia among pregnant women attending ANC in a University hospital in Eastern Ethiopia, 2021 (*n* = 442).

		**Anemia**			
**Characteristics**	**Category**	**Yes, *n* (%)**	**No, *n* (%)**	**COR (95 % CI)**	**AOR (95 % CI)**
Birth interval	<2 years	51 (72.9)	19 (27.1)	6.05 (3.45, 7.34)^**^	3.24 (1.88, 4.32)^**^
	≥ 2 years	61 (27.5)	161 (72.5)	1.00	1.00
Number of children	≤ 1 child	56 (20.3)	220 (79.7)	1.00	1.00
	≥ 2 children	56 (33.7)	110 (66.3)	4.12 (2.68, 8.77)^**^	2.54 (1.12–4.64)^*^
Residence	Urban	79 (23.0)	265 (77.0)	1.00	1.00
	Rural	33 (33.7)	65 (66.3)	2.12 (1.43, 3.18)^*^	1.89 (0.18, 3.04)
Monthly income	<1000 birr	30 (32.3)	63 (67.7)	4.43 (1.65, 5.70)^**^	2.89 (1.31, 5.58)^*^
	1000–2000 birr	34 (29.6)	81 (70.4)	1.38 (0.67, 2.89)	1.16 (0.49, 2.74)
	>2000 birr	48 (20.5)	186 (79.5)	1.00	1.00
Educational level	Not educated	15 (33.3)	30 (66.7)	1.76 (1.6, 5.2)^*^	0.1.10 (0.67, 2.74)
	Primary school	63 (25.3)	186 (74.7)	0.82 (0.53, 1.20)	0.21 (0.03, 0.19.3)
	Secondary and above	34 (23.0)	114 (77.0)	1.00	1.00
Gravida	0–1	12 (8.6)	128 (91.4)	1.00	1.00
	≥2	100 (33.1)	202 (66.9)	1.01 (1.46,3.95)^*^	0.96 (0.14, 2.81)
Trimester of pregnancy	1^st^ trimester	13 (16.9)	64 (83.1)	1.00	1.00
	2^nd^ trimester	79 (24.9)	238 (75.1)	1.4 (1.38, 5.21)^*^	1.63 (0.19, 3.65)
	3^rd^ trimester	20 (41.7)	28 (58.3)	6.32 (6.98,14.69)^**^	3.87 (4.86, 12.62)^*^
Menstrual disorder	Yes	16 (53.3)	14 (46.7)	2.43 (1.02,16.26)^**^	2.28 (1.69, 5.24)^*^
	No	96 (23.3)	316 (76.7)		1.00
History of PPH	Yes	4 (50.0)	4 (50.0)	1.40 (1.99, 4.28)^*^	1.32 (0.74–9.06)
	No	108 (24.9)	326 (75.1)	1.00	
History of abortion	Yes	38 (50.7)	37 (49.3)	0.67 (0.26, 0.72)^*^	1.22 (0.61, 2.44)
	No	64 (17.4)	293 (82.6)	1.00	1.00
Drink coffee/tea within 30 min	Yes	97 (29.6)	231 (70.4)	2.18 (1.38, 7.29)^*^	1.03 (0.88, 2.06)
	No	15 (13.2)	99 (86.8)	1.00	1.00
Chewing chat	Yes	35 (41.2)	50 (58.8)	1.67 (1.18, 2.30)^*^	1.01 (0.79, 8.35)
	No	77 (21.6)	280 (78.4)	1.00	1.00

** *p < 0.001*,

* *p < 0.05*.

## Discussion

This study assessed the magnitude anemia and its associated factors among the pregnant women who were attending antenatal care in Hiwot Fana specialized university hospital in eastern Ethiopia. It revealed that one in four pregnant women was anemic. Our prevalence rate for anemia in pregnant women in eastern Ethiopia is high enough for anemia to be classified as a moderate public health problem according to WHO (37). Women who had a birth interval of <2 years, who had two or more children, who had monthly income of <1,000 birr, whose gestation age was in third trimester, and who had abnormal menstrual history were significantly associated with anemia.

According to the findings of this study, the overall magnitude of anemia among pregnant women was (25.3%) [95%CI: (21.5–29.2%)]. A similar finding was reported in a study conducted in Mizan Tepi (23.5%) (38), North west Ethiopia (25.2%) (8), and Gonder (23.2%) (39). On the other hand, this finding was relatively lower than studies conducted in Arab Minch (32.8%) (10), eastern Ethiopia (33.1%) (40), Western Arsi (36.6%) (41), Nejo (37.8%) (22), Woldia (39.1%) (42), Turkey (41.6%) (43), Ghana (50.8%) (32), Gode Town (56.8%) (5), Bodit town 61.6%) (10), Gulu district (65.4%)(12), India (81.5%) (26), and Nepal (82.6%) (27). However, it is higher than the studies conducted in Addis Ababa (11.6%) (15) and Adama (14.9%) (9). These differences could be attributed to the differences in socioeconomic characteristics, study settings and periods, dietary habits, and health-seeking behaviors between towns with a community of relatively different lifestyle, feeding practices, and social norms.

This study pointed out that a pregnant woman with a birth interval of <2 years were more likely to become anemic than those with a birth interval of more than 2 years. This finding is consistent with the findings of a Saudi Arabian study (44), Arba Minch (10), and southern Ethiopia (23). This might be related to decreased iron store of women due to the occurrence of pregnancy in quick succession between subsequent pregnancies. This emphasizes the importance of birth spacing using any appropriate contraceptive method to prevent anemia in pregnant women. As a result, concerned bodies should work hard to disseminate health education that enables reproductive-age women to avoid becoming pregnant with birth intervals of <2 years. Because she needs to rest her uterus and replenish her iron stores before becoming pregnant.

According to this study, the likelihood of anemia among pregnant women who have monthly income < 1,000 birr was almost three times more likely to have anemia than their counter parts. This is supported by the studies conducted in Turkey (43), Wolayita (29), Arba Minch (10), and Addis Ababa (15). This could be because women from lower socioeconomic backgrounds are unable to purchase high-quality foods which are the source of iron in sufficient quantities, putting them at risk of anemia.

It is generally believed that anemia in pregnancy increases with rising parity, due to repeated drain on iron stores (45). Pregnant women who have ≥ 2 children were two and half times more likely to have anemia than women who have ≤ 1 child. This is in harmony with the studies conducted in Turkey (43), Arba Minch (10), Wolayita (23), and Gonder (46). This could be due to the effect of blood loss during each delivery and the difficulty in obtaining adequate foods for many family sizes. Preconception care, such as the provision of contraception, is critical in preventing anemia during pregnancy (47).

In this study, being a third trimester pregnancy was nearly four times more likely to have anemia compared to first trimester pregnancy. This finding is consistent with a study done in Saudi Arabia (48), Turkey (43), Northern Ghana (49), Tigray (50), Hawasa (33), and Addis Ababa (7). Additionally, studies conducted in Malaysia, Vietnam, and Nepal found that increased gestational age is significantly associated with the risk of developing anemia (51–53). This could be due to the fact that when the gestational age increases, the mother becomes weak and the iron in the blood is shared with the fetus in the womb therefore decreasing the iron-binding capacity of the mother's blood. Furthermore, the need for calories and nutrients increases during pregnancy to support increased maternal metabolism, blood volume, and nutrient delivery to the fetus (54, 55), and this demand increases during the third trimester of pregnancy. There is a significant decrease in iron absorption during the first trimester, most likely due to lower iron requirements and the cessation of menstruation, saving a median of 0.56 mg iron/day (160 mg/pregnancy) (54, 56). However, iron absorption from a diet with very high iron bioavailability increases by 1.9 mg/day in the second trimester and by up to 5.0 mg/day in the third trimester (57).

This study pointed out that pregnant women who have history of abnormal menstrual history were two times more likely to have anemia than their counter parts. This is supported by study done in southern Ethiopia (23), Mekelle city (58), Dessie Hospital (35), and Mizan Tepi (38). This could be due to iron deficiency anemia, which is one of the side effects of excessive menstrual loss. All menstruating women, including adolescents, should receive iron (60 mg) and folic acid (2.8 mg) supplements weekly on a regular basis in communities where iron deficiency anemia (IDA) is a problem.

The study's strength was that it investigated a problem that was frequently ignored by healthcare workers and that it was a representative because it covered a larger geographical area. On the other hand, because this study was cross-sectional, it cannot identify causation, and the sampling method included only clients who had no ANC visit for the current pregnancy. Furthermore, the study's limitation was the inability to recall past food intake, which could have resulted in measurement.

## Conclusion

In this study, approximately one in every four pregnant women was anemic. Birth intervals, number of children, history of menstrual disorder, monthly income, and trimester of pregnancy were the factors significantly associated with anemia in pregnant women.

Majority of predictors of anemia are modifiable in nature. Averting anemia in pregnant women necessitates increasing contraceptive use, preconception care, improving birth intervals, and iron consumption before and during pregnancy.

## Data Availability Statement

The raw data supporting the conclusions of this article will be made available by the authors, without undue reservation.

## Ethics Statement

The studies involving human participants were reviewed and approved by IHRERC, Haramya University. The patients/participants provided their written informed consent to participate in this study.

## Author Contributions

The conception of the work, design of the work, acquisition of data, analysis, and the interpretation of the data were carried out by BB and AD. Data curation, drafting the article, critically reviewing it for intellectual content, validation, and final approval of the version to be published were done by BB, AD, YD, AE, HB, BE, DT, BN, TG, SM, SH, BTM, AA, and TAY. All authors read and approved the final manuscript.

## Funding

Haramaya University provided the financial supports for this study. But the funding agency had no role in the collection, analysis, and interpretation of the data as well as the writing-up of the manuscript.

## Conflict of Interest

The authors declare that the research was conducted in the absence of any commercial or financial relationships that could be construed as a potential conflict of interest.

## Publisher's Note

All claims expressed in this article are solely those of the authors and do not necessarily represent those of their affiliated organizations, or those of the publisher, the editors and the reviewers. Any product that may be evaluated in this article, or claim that may be made by its manufacturer, is not guaranteed or endorsed by the publisher.
